# Vitamin D Supplementation and Mental Health in Multiple Sclerosis Patients: A Systematic Review

**DOI:** 10.3390/nu13124207

**Published:** 2021-11-24

**Authors:** Dominika Głąbska, Aleksandra Kołota, Katarzyna Lachowicz, Dominika Skolmowska, Małgorzata Stachoń, Dominika Guzek

**Affiliations:** 1 Department of Dietetics, Institute of Human Nutrition Sciences, Warsaw University of Life Sciences (WULS-SGGW), 159C Nowoursynowska Street, 02-776 Warsaw, Poland; aleksandra_kolota@sggw.edu.pl (A.K.); katarzyna_lachowicz@sggw.edu.pl (K.L.); dominika_skolmowska@sggw.edu.pl (D.S.); malgorzata_stachon@sggw.edu.pl (M.S.); 2 Department of Food Market and Consumer Research, Institute of Human Nutrition Sciences, Warsaw University of Life Sciences (WULS-SGGW), 159C Nowoursynowska Street, 02-776 Warsaw, Poland; dominika_guzek@sggw.edu.pl

**Keywords:** multiple sclerosis, mental health, vitamin D, supplement, supplementation, quality of life, depression, depressive symptoms, fatigue

## Abstract

Vitamin D has a promising role in multiple sclerosis (MS) management, and it has been found to be beneficial for patients’ mental health, which is reduced in MS patients. The aim of the present study was to conduct a systematic review of the literature to assess the influence of vitamin D supplementation on mental health in MS patients. The systematic review was registered in the PROSPERO database (CRD42020155779) and it was conducted on the basis of the PRISMA guidelines. The search procedure was conducted using PubMed and Web of Science databases and it included studies published up until September 2021. Six studies were included in the systematic review. The risk of bias was analyzed using the Newcastle–Ottawa Scale (NOS). Within the included studies, there were two studies randomized against placebo and four other prospective studies. The studies presented vitamin D interventions randomized against placebo or not randomized, while supplementation was applied for various durations—from 4 weeks to 12 months, or the studies compared patients who applied vitamin D supplementation and those who did not apply it and verified the effect of the supplementation after a number of years. The mental health outcomes that were assessed included quality of life, depression/depressive symptoms, and fatigue as an additional element. The majority of studies supported the positive influence of vitamin D on the mental health of MS patients, including the study characterized as having the highest quality (randomized against placebo with the highest NOS score). All the studies that assessed the quality of life indicated the positive influence of vitamin D while the studies that did not find a positive influence of vitamin D were conducted for depression/depressive symptoms. In spite of the fact that only a small number of studies have been conducted so far, and only two studies were randomized against a placebo, some conclusions may be formulated. The systematic review allowed us to conclude that there may be a positive effect of vitamin D supplementation in MS patients, which was stated in all of the studies analyzing quality of life, as well as in one study analyzing depressive symptoms. Considering that vitamin D deficiency is common in MS patients, and the potential positive influence of supplementation on the quality of life, supplementation should be applied at least in doses that cover the recommended intake.

## 1. Introduction

Multiple sclerosis (MS) is defined as an immune-mediated disease of the central nervous system [1], which is characterized by chronic inflammation, demyelination, gliosis, and neuronal loss, leading to physical or cognitive disability and neurological defects [2]. There are several subtypes of MS, while the most common is relapsing remitting MS (about 87% of MS patients are diagnosed with this subtype), which is characterized by alternating periods of remission and unpredictable attacks [3].

The prevalence of MS is increasing in all regions of the world and in 2020 the number of affected individuals was estimated as 2.8 million worldwide (with a global frequency of 35.9 per 100,000 individuals) [4]. The typical symptoms of MS include weakness or numbness in one or more limbs; optic neuritis, associated with painful monocular visual loss; tremor and ataxic gait from cerebellar dysfunction; double vision, dysarthria, or dizziness from brainstem dysfunction; and fatigue [5]. At the same time, MS is also associated with other potential consequences, for example, a systematic review and meta-analysis by Hong et al. [6] indicated the increased risk of developing any type of stroke, and especially, the increased risk of developing ischemic stroke. Taking this into account, MS is associated with severe disabilities resulting in a reduction in life expectancy of 7–14 years [7].

However, not only physical consequences are observed; the systematic review by Neuhaus et al. [8] proved that among other cognitive measures, the decision-making abilities of MS patients may be impaired, which may influence their everyday life. The prevalence of depression in MS is also higher than for the general population, by even up to 60% depending on the group studied. [9]. Similarly, the quality of life in MS patients is reduced when compared with the general population [10]. The systematic review by Gil-González et al. [11] revealed that MS is associated mainly with disability, fatigue, depression, cognitive impairment and unemployment. Such a situation causes an increased risk of suicide in MS, and its frequency is twice as high as that for the general population [12,13].

Taking into consideration the course of MS, it is emphasized that adequate management of the disease should include not only palliative care, but also a dedicated approach to improve life expectancy and quality of life [14]. A dietary approach is among various possible actions that are indicated, as some dietary modifications may influence the course of the disease and resultant quality of life. Various dietary factors, including vitamin D, have been linked with MS incidence and course, thus they are indicated as a potentially influencing factors [15].

Low blood levels of 25-hydroxyvitamin D (25(OH)D) are associated with increased risk of MS [16] and in the early disease course it is a strong risk factor for long-term MS activity and progression [17]. As vitamin D receptors are present in almost every immune cell, while the active form of vitamin D is crucial for lymphocyte regulation and cells proliferation, vitamin D has been linked with a predisposition to autoimmune diseases [18]. Furthermore, numerous environmental factors linked to MS incidence may also alter vitamin D levels. The susceptibility to these environmental risk factors may be modulated by genetic variation and epigenetic mechanisms, such as DNA methylation, histone modification, and ncRNA [19].

The systematic review and meta-analysis by Martínez-Lapiscina et al. [20] indicated that serum 25(OH)D levels are associated with a modest decrease in the relapse rate and radiological inflammatory activities in patients with MS. However, a systematic review and meta-analysis of randomized controlled trials by Doosti-Irani et al. [21] indicated that vitamin D supplementation in MS had no significant effect on the expanded disability status scale (EDSS) and the meta-analysis by Zheng et al. [22] indicated that it had no significant effect on EDSS, or on annual relapse rate. Based on the gathered evidence, it was concluded in a comprehensive review by Sintzel et al. [23], that the impact of vitamin D supplementation on MS activity remains inadequately investigated. Moreover, there is a knowledge gap in regard to chronic and high-dose therapy, which can lead to life-threatening complications related to vitamin D toxicity in this group [24]. 

It has been suggested that vitamin D plays a role not only in the physical health, but also the mental health of healthy individuals. It has been indicated for reducing the risk of depression, as concluded by the meta-analyses of Vellekkatt and Menon [25], Shaffer et al. [26], and Spedding [27], as well as for reducing negative emotions, as concluded in the meta-analysis of Cheng et al. [28]. Taking into account the promising role of vitamin D in MS management, as well as the mental health problems of MS patients, the aim of the present study was to conduct a systematic review of the literature to assess the influence of vitamin D supplementation on mental health in MS patients.

## 2. Materials and Methods

### 2.1. The Systematic Review Registration and Design

The systematic review was registered in the International Prospective Register of Systematic Reviews (PROSPERO) (CRD42020155779). Systematic reviews to assess the influence of vitamin D on mental health in children [29], as well as in adults with diabetes [30] and in adults with inflammatory bowel diseases and irritable bowel syndrome were conducted [31] within the project.

The systematic review was conducted on the basis of the Preferred Reporting Items for Systematic Reviews and Meta-Analyses (PRISMA) guidelines [32]. The search procedure was conducted using PubMed and Web of Science databases and it included all studies published in English, as full-texts in peer-reviewed journals. The search was conducted in the following stages: (1) a search within studies published up until October 2019 (as described in the previous study [29]), and (2) a search within studies published from October 2019 to September 2021 (as described in the previous studies [30,31]).

### 2.2. Criteria for Including Studies in a Systematic Review

The search was conducted in order to select studies analyzing the influence of any vitamin D supplementation on any mental health outcome in adult MS patients. The subtype and course of MS, as well as the country or population, were not defined in order to include all the studies assessing the studied influence.

The inclusion criteria were formulated as follows:

(1) Population of adults;
(2) Confirmed diagnosis of MS;
(3) Any vitamin D supplementation applied;
(4) Any mental health outcome assessed (including subjective and/or objective measures).

The exclusion criteria were formulated as follows:

(1) Studies in animal models;
(2) Confirmed concurrent diagnosis of any intellectual disabilities;
(3) Confirmed concurrent diagnosis of any eating disorders;
(4) Confirmed concurrent diagnosis of any other neurological disorders, changing dietary behaviors (e.g., Alzheimer’s disease, epilepsy);
(5) Vitamin D applied within multiple nutrients supplementation.

The summary of the patient, intervention/exposure, comparator, outcome, and study design (PICOS) criteria for inclusion and exclusion of studies to a systematic review are presented in Table 1.

### 2.3. The Search and Inclusion Procedure

The electronic search strategy applied for the systematic review of PubMed and Web of Science databases is presented in the Supplementary Materials, Table S1.

The search procedure was followed by removing duplicated records and identifying potentially eligible studies, which was based on the established inclusion criteria and exclusion criteria. The identification of potentially eligible studies was conducted in 3 phases, which were based on title screening, abstract screening and full texts screening. Titles and abstracts were based on PubMed and Web of Science databases, and if studies were defined as potentially eligible, the full texts were obtained. If full texts were not available, the corresponding authors were contacted to obtain them. The assessment of titles, abstracts and full texts was conducted independently by 2 researchers and in case of any disagreement, the third researcher participated.

**Figure 1:**
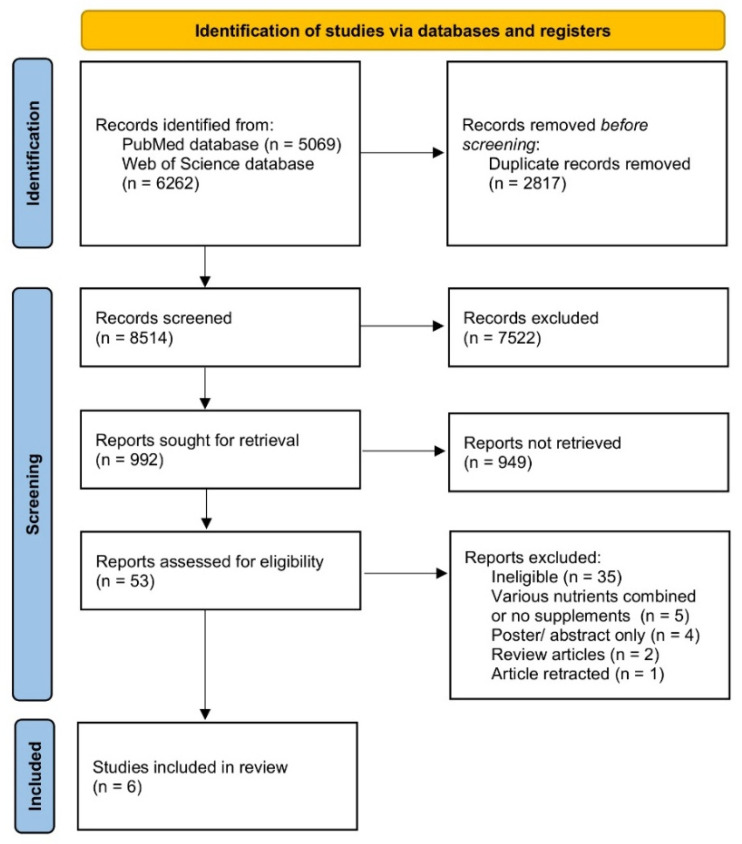
The procedure of identification, screening, eligibility assessment and inclusion of studies in the systematic review.

The process of identification, screening, eligibility assessment and the inclusion of studies in the systematic review is presented in Figure 1.

### 2.4. Data Extraction and Studies Assessment Procedure

The included studies were used to obtain the data necessary to describe the observed influence of vitamin D supplementation on mental health outcomes. The extracted data included:

(1) The general presentation of the studies included and of the populations assessed within (authors and year; study design; country/location; population assessed; time);
(2) The description of the studied group (number of participants; number of female participants; age; inclusion criteria; exclusion criteria);
(3) The description of the vitamin D supplementation/intervention (vitamin D measure and dosage regimen) and of the mental health outcome (assessed outcome and psychological measure);
(4) The observations and conclusions.

The data were obtained from the published version of the manuscript, but if such information was not presented, the corresponding authors were contacted to obtain them (such data are within the study presented as provided on request). The data extraction was conducted independently by 2 researchers and in case of any disagreement, the third researcher participated.

The quality of the included studies was assessed while using the Newcastle–Ottawa Scale (NOS) dedicated to non-randomized studies [33]. The included studies were assessed within the criteria for selection (scored from 0 to 4), comparability (scored from 0 to 2) and exposure/outcome (scored from 0 to 3). The final score was attributed to a specific category of very high risk of bias (total scored from 0 to 3), high risk of bias (total scored from 4 to 6) and low risk of bias (total scored from 7 to 9), as it is commonly applied [34].

## 3. Results

The design and basic details of the studies of MS patients included in the systematic review [35,36,37,38,39,40] are presented in Table 2. Of the included studies, there were two studies randomized against placebo [35,36] and four other prospective studies [37,38,39,40]. They were conducted in international cohorts [39,40], as well as specific countries—Iran [35], The Netherlands [36], and Saudi Arabia [37]. There were studies conducted in populations of patients with relapsing remitting multiple sclerosis [35,36,37], and studies conducted in populations of patients with an undefined subtype of MS [38,39,40].

The characteristics of the MS patients assessed within the studies included in the systematic review are presented in Table 3. The included studies presented observations formulated in samples of various sizes—small samples of less than 50 participants [36,37], medium size sample of about 100 participants [35,38], or a large international sample [39,40]. The majority of studies were conducted in participants of about 30–40 years [35,36,37,38], but the largest international study included older participants of age almost 50 years old [39,40]. The majority of studies included patients with MS diagnosed based on McDonald criteria [35,36,37,38], sometimes accompanied by magnetic resonance imaging (MRI) results [36,37], but the largest international study was based on the self-reported information about MS diagnosis [39,40].

The description of the vitamin D exposures applied within the studies and mental health outcomes assessed within the studies of MS patients included in the systematic review is presented in Table 4. The majority of studies presented defined vitamin D intervention in a population randomized against placebo [35,36] or not randomized [37,38], which were applied for various durations—from 4 weeks [36] to 12 months [37]. However, the largest international study [39,40] did not present a planned intervention, but compared patients declaring they applied vitamin D supplementation and those who did not declare it, and it verified the effect of the supplementation after 2.5 years. The mental health outcomes that were assessed, included quality of life [35,38,40], and depression/depressive symptoms [36,37,39], but also fatigue as an additional element [36,38].

The observations and conclusion formulated within the studies of MS patients included in the systematic review are presented in Table 5. All the included studies indicated some positive association between vitamin D supplementation and the mental health of MS patients, but in some studies, they were observed only in some of the analysis or only for some aspects of mental health. Even if the authors of the study concluded there was no evidence for a reduction in depressive symptoms upon vitamin D supplementation, it should be noted that within the study they observed a significant decrease in Hospital Anxiety and Depression Scale depression subscale (HADS-D) scores in a group receiving vitamin D supplements, but only a trend towards a decrease within a placebo group [36]. Similarly, in a study that indicated no association between vitamin D supplementation and depression risk, such a situation was observed after adjusting for potential confounders, but the authors observed an existing association before the adjustment [39].

A summary of the conclusions from the studies of MS patients included in the systematic review accompanied by the assessment of the quality of studies based on the design of the studies and the NOS score is presented in Table 6. The majority of studies supported the positive influence of vitamin D on the mental health of MS patients [35,37,38,40], including the study characterized as having highest quality (study randomized against placebo with the highest NOS score) [35]. However, among two studies that did not support the positive influence of vitamin D [36,39], one study was also randomized against placebo [36]. All the studies assessing the quality of life indicated the positive influence of vitamin D [35,38,40], while the studies that did not find a positive influence of vitamin D were conducted for depression/depressive symptoms [36,39].

## 4. Discussion

In spite of the fact that only a small number of studies have assessed the effect of vitamin D supplementation on mental health in MS patients, those that were gathered indicate the potential positive influence of such supplementation. In particular, the results of the prospective intervention-based studies should be taken into account [35,36,37,38,39,40], including the results of the studies randomized against placebo [35,36]. Moreover, it should be noted that the studies based on the self-reported vitamin D supplementation, not being applied within an intervention study [39,40], were elaborated based on the results of the same Health Outcomes and Lifestyle In a Sample of people with Multiple Sclerosis (HOLISM) Study and the sample size was identical in two studies, thus it may be supposed that the studies present the same group that were analyzed separately for the influence on depression [39] and quality of life [40]. Taking this into account, it may be suggested that in this studied group, the beneficial effect of the applied supplementation was confirmed for the quality of life [40], but was not confirmed for depression [39]. As a result, it cannot be stated that two studies did not confirm any positive effect of vitamin D supplementation for the mental health of MS patients, but only one [36], as the other study, namely, the HOLISM Study, confirmed this effect but only for one assessed mental health outcome—quality of life [40].

The gathered observations indicated that quality of life was a mental health outcome influenced by vitamin D supplementation in MS patients in all the presented studies [35,38,40]. Quality of life is defined by the World Health Organization (WHO) as individuals’ perceptions of their position in life in the context of the culture and value systems in which they live and in relation to their goals, expectations, standards and concerns. This definition includes not only physical health, or level of independence (both important for MS patients), but also psychological state, social relationships, and personal beliefs [41]. While clinicians focus mainly on the physical aspects of quality of life, MS patients emphasize the role of the mental health aspects of the quality of life [42], and consider that vitality, pain, as well as general, emotional, and mental health are also relevant [43], which confirms the role of quality of life within general mental health. In MS patients the quality of life is significantly decreased compared to healthy individuals [44] and this is indicated within the factors associated with the disease progression [45]. Taking this into account, the possibility of improving the quality of life of MS patients by applying vitamin D supplementation is of a great value.

Additionally, for depressive symptoms [37], one study in this systematic review indicated a beneficial influence, so depressive symptoms may also be potentially influenced by vitamin D supplementation. However, the influence of vitamin D supplementation was not studied for other mental health outcomes that are also influenced by MS, such as well-being [46], sleep quality [47], life satisfaction [48], self-efficacy and self-esteem [49], optimism [50], stress [51], mood disorders [52], anxiety [53], psychiatric syndromes [54], and suicidal ideation [55].

Vitamin D is indicated as a factor associated with MS incidence [16] and progression [17,20], but it is emphasized that high doses of vitamin D generate a risk of toxicity in this group [24]. However, it should be also noted that vitamin D deficiency in the general population is a global public health problem in all age groups [56], and due to the frequency of this deficiency and its role in the treatment of many diseases, it is indicated as an important, inexpensive, and safe adjuvant therapy for many diseases [57]. Additionally, in case of MS, patients generally have a low vitamin D dietary intake [58], and as a result, a low 25(OH)D blood level [59,60]. Taking this into account, in case of documented vitamin D deficiency, its supplementation is recommended to replenish vitamin D stores [61], thus it should be applied to MS patients with documented vitamin D deficiency. However, based on the risk of its toxicity [24], the doses should firstly meet the general dietary intake recommendations [62]. The second-line target should be based on the potential benefits of increased vitamin D supply, in association with the course of MS [17,20], but also, as indicated in the presented systematic review, in association with the patient’s mental health [35,37,38,40]. However, this area needs more studies that are randomized against placebo. Based on the conducted systematic review of the influence of vitamin D supplementation on the mental health of MS patients, supplementation should be applied at least in doses that cover the recommended intake and further research is needed to define the level required to obtain additional benefits. However, to deepen our knowledge, not only the association between vitamin D supplementation and mental health should be studied, but also the potential further consequences associated with MS symptoms or course.

## 5. Conclusions

This systematic review of the literature was conducted to assess the influence of vitamin D supplementation on mental health in MS patients. Based on this review, we concluded that there may be a positive effect of vitamin D supplementation. Such a positive effect was found in all the studies analyzing quality of life, as well as in one study that analyzed depressive symptoms. Taking into consideration that vitamin D deficiency is common in MS patients, and the potential positive influence of supplementation on the quality of life in this group, supplementation should be applied at least in a dose that covers the recommended intake.

## Figures and Tables

**Table 1:** The summary of the patient, intervention/exposure, comparator, outcome, and study design (PICOS) criteria for inclusion and exclusion of studies in a systematic review.

PICOS Parameter	Inclusion Criteria	Exclusion Criteria
Population	Multiple sclerosis adult patients	Children and adolescents with multiple sclerosis, individuals with any intellectual disabilities, any eating disorders, or any other neurological disorders
Intervention/exposure	Any vitamin D supplementation applied	Vitamin D applied within multiple nutrients supplementation
Comparison	Influence on a mental health outcomes assessed while compared with baseline/placebo/various doses and regimens	Lack of comparison
Outcome	Any mental health outcome assessed	Cognitive function assessed
Study design	Articles published in peer-reviewed journals, in English	Articles not published in English, reviews, meta-analyses, expert opinions, letters to editor, comments, studies in animal models, methodological articles, case reports, conference reports

**Table 2:** The design and basic details of the studies of multiple sclerosis (MS) patients included to the systematic review.

Ref.	Authors, Year	Study Design	Country/Detailed Location	Study Group	Time
[35]	Ashtari et al., 2016	Randomized, double-blind, placebo-controlled clinical trial	Iran	Patients with relapsing remitting multiple sclerosis	22 December 2013–June 2014 *
[36]	Rolf et al., 2017	Randomized placebo-controlled pilot study within SOLARIUM sub-study of the SOLAR trial	The Netherlands	Patients with relapsing remitting multiple sclerosis recruited in four hospitals in The Netherlands from SOLARIUM sub-study of the SOLAR trial	February 2011–April 2015 *
[37]	Kotb et al., 2019	Prospective cross-sectional observational study	Saudi Arabia/Alkharj	Patients with relapsing remitting multiple sclerosis from Prince Sattam Bin-Abdulaziz University Hospital	2013–2018
[38]	Beckmann et al., 2020	Prospective study	Not specified	Patients with multiple sclerosis from multiple sclerosis outpatient clinic	2016–2017
[39]	Taylor et al., 2018	Longitudinal prospective study within Health Outcomes and Lifestyle In a Sample of people with Multiple Sclerosis (HOLISM) Study	Participants living in 57 countries, while 88% resided in the United States, Australia, United Kingdom, New Zealand, and Canada	Adults with multiple sclerosis from HOLISM Study	2012–2015 *
[40]	Simpson-Yap et al., 2021	Longitudinal prospective study	International cohort	Adults with multiple sclerosis from HOLISM Study	2012–2015 *

* Data provided on request.

**Table 3:** The characteristics of the multiple sclerosis (MS) patients assessed within the studies included in the systematic review.

Ref.	Number of Participants (Number of Females)	Age (Mean Years with SD)	Inclusion Criteria/Exclusion Criteria
[35]	94 (80)	31.4 ± 7.6 for vitamin D supplementation group 34.6 ± 10.1 for placebo group	Inclusion: aged 18–55 years; definite diagnosis of relapsing remitting multiple sclerosis according to McDonald criteria; EDSS score <4; no relapse 30 days before inclusion; negative β-HCG test for women in child-bearing age Exclusion: pregnancy; lactation; any disease other than MS; 25(OH)D serum level >85 ng/mL; past history of renal or hepatic disease; relapse during the study; received corticosteroids in the previous 30 days; calcium >11 mg/dL; aspartate transaminase or alanine transaminase >3 times normal values; alkaline phosphatase >2.5 times normal values
[36]	40 (26)	38.5 ± 7.8 for vitamin D supplementation group 37.6 ± 9.6 for placebo group	Inclusion: aged 18–55 years; diagnosed with relapsing remitting multiple sclerosis according to the original or 2005 revised McDonald criteria confirmed by MRI; treated with interferon-β1α; first clinical event in the previous 5 years; active disease in the previous 18 months, but not in the 30 days prior to inclusion Exclusion: use of oral or systemic glucocorticoids or ACTH within 30 days prior to inclusion; a history or presence of severe depression; a history of suicide attempt or current suicidal ideation; current or past drug or alcohol abuse; missing data
[37]	35 (19)	27.0 ± 4.0	Inclusion: aged ≥18 years; relapsing remitting multiple sclerosis according to McDonald criteria; no exacerbations; no gadolinium enhancing lesions on MRI; no corticosteroid therapy within four weeks prior to recruitment; regular treatment with interferon-β Exclusion: treatment other than interferon; high-dose of vitamin D (daily intake of at least 25 µg) before inclusion to the study; immunomodulatory therapy changed within the past 3 months; history of systemic glucocorticoid therapy or relapse within 30 days; severe depression; pregnancy; serum creatinine >1.5 mg/dL; hypersensitivity to vitamin D preparations; history of hyperparathyroidism, tuberculosis, sarcoidosis, or nephrolithiasis
[38]	149 (107)	37.52 ± 9.82	Inclusion: diagnosed with multiple sclerosis according to 2010 revised McDonald criteria Exclusion: disorders related to vitamin D deficiency (e.g., parathyroid pathologies); other acute or chronic disease at time of blood sampling determined by routine tests; receiving vitamins (vitamin D or multivitamin compounds) as supplements in the 6 months preceding data collection; relapse in the last 30 days; pregnancy; breastfeeding; other neurological or immune-mediated disease; skin diseases; medication use with a medical recommendation to avoid exposure to the sun; applying hydrochlorothiazide, barbiturates, phenytoin or digitalis
[39]	1401 (1150)	48.4 ± 10.5	Inclusion: aged ≥18 years, diagnosed with multiple sclerosis (MS) by a medical doctor Exclusion: missing data *
[40]	1401 (1152)	48.4 ± 10.5	Inclusion: aged ≥18 years, diagnosed with multiple sclerosis (MS) by a medical doctor Exclusion: missing data *

* Data provided on request; ACTH—adrenocorticotropic hormone; β-HCG—beta subunit of human chorionic gonadotropin; EDSS—expanded disability status scale; MRI—magnetic resonance imaging.

**Table 4:** The description of the vitamin D exposures applied within the studies and mental health outcomes assessed within the studies of multiple sclerosis (MS) patients included to the systematic review.

Ref.	Vitamin D Measure	Assessed Vitamin D Supplementation	Mental Health Outcome	Psychological Measure
[35]	25(OH)D blood level	1250 µg/5 days for 3 months vs. placebo	Quality of life	Multiple sclerosis quality of life (MSQOL-54)—Persian version
[36]	25(OH)D blood level	175 µg/day for 4 weeks followed by 350 µg/day for 44 weeks vs. placebo	(1) Depressive symptoms (2) Fatigue	(1) Depression subscale of the Hospital Anxiety and Depression Scale (HADS) (2) Dutch version of the Fatigue Severity Scale (FSS)
[37]	25(OH)D blood level	250 µg/day for 12 months	Depressive symptoms	Beck’s depression inventory (BDI)
[38]	25(OH)D blood level	In patients with serum 25(OH)D <30 ng/mL–1250 µg/week for 8 weeks (to reach a minimum serum 25(OH)D level of 30 ng/mL) followed by 37.5–50 µg/day In patients with serum 25(OH)D of 20–30 ng/mL–37.5–50 µg/days	(1) Quality of life (2) Fatigue	(1) Multiple sclerosis-related quality of life inventory (MSQOLI) (2) Fatigue Severity Scale (FSS)
[39]	Taking vitamin D supplement	Declared vitamin D supplementation taken vs. not taken in the follow up of 2.5 years	Depression	Patient Health Questionnaire-2 (PHQ-2) Patient, Health Questionniare-9 (PHQ-9)
[40]	Taking vitamin D supplement	Declared vitamin D supplementation taken vs. not taken in the follow up of 2.5 years	Quality of life	Multiple sclerosis quality of life (MSQOL-54)

**Table 5:** The observations and conclusion formulated within the studies of multiple sclerosis (MS) patients included to the systematic review.

Ref.	Observations	Conclusions
[35]	After 3 months, the vitamin D group had a significant difference in mental health component of quality of life with placebo group, 62.41 ± 13.99 vs. 60.99 ± 17.99 (*p* = 0.041). Change in health component of quality of life was 75.74 ± 25.73 and 70.59 ± 26.45 in vitamin D and placebo group, respectively (*p* = 0.036).	Mental quality of life improved significantly after taking high dose vitamin D for 3 months in vitamin D group relative to placebo. Also a positive change in health status was reported by patients receiving high dose vitamin D relative to placebo group.
[36]	Pre- and post-supplementation depression scores, measured using the Hospital Anxiety Depression Scale (HADS) depression subscale (HADS-D), showed a significant decrease within the vitamin D3 group (median HADS-D 4.0 to 3.0, *p* = 0.02), a trend towards a decrease within the placebo group (median HADS-D 3.0 to 2.0, *p* = 0.06), but no significantly different reductions between groups (*p* = 0.78).	There was no evidence for a reduction of depressive symptoms upon vitamin D3 supplementation in relapsing remitting multiple sclerosis patients.
[37]	Depressive symptoms were high at baseline and improved with vitamin D replacement although, Expanded Disability Status Scale (EDSS) score was not improving. Vitamin D levels correlated negatively with depressive symptoms at baseline and follow up periods.	Lower vitamin D levels are associated with higher depressive scores, and vitamin D replacement could improve depressive symptoms in patients with relapsing remitting multiple sclerosis.
[38]	After vitamin D supplementation, health-related quality of life and fatigue scores improved significantly. There was a direct association between health-related quality of life with absence of fatigue and vitamin D status at the end of study.	The 90% frequency of multiple sclerosis patients with vitamin D deficiency, together with the significant association of vitamin D status with the absence of fatigue and improved physical and functional well-being, points to vitamin D supplementation as a potential therapy to enhance the patient’s quality of life.
[39]	Vitamin D supplementation at baseline was associated with lower frequency of positive depression-screen 2.5 years later. After adjusting for potential confounders, vitamin D supplementation was not associated with a change in risk for depression.	Vitamin D supplementation was associated with lower frequencies of depression risk, but this association was no longer significant after adjusting for potential confounders.
[40]	At 2.5-year follow-up, quality of life scores were higher among participants reporting taking vitamin D supplements (physical: aβ = 3.58, 95% CI = 1.35–5.80; mental: aβ = 3.08, 95% CI = 0.72–5.44), particularly average daily dose over 125 µg/d. Baseline-reported vitamin D supplementation was associated with greater increase in physical (aβ = 1.02, 95% CI = 0.22–1.81), but not mental quality of life (aβ = 0.11, 95% CI = −1.00–1.23).	Self-reported vitamin D supplement use was cross-sectionally associated with higher physical and mental quality of life, but prospectively only with increased physical quality of life.

CI—confidence interval.

**Table 6:** The summary of conclusions from the studies of multiple sclerosis (MS) patients included in the systematic review accompanied by the assessment of the quality of studies based on the design of the studies and the Newcastle-Ottawa Scale (NOS) score.

Ref.	Studied Outcome	Conclusion about General Influence of Vitamin D on Mental Health ^a^	Quality of the Study Based on the Study Design ^b^		Quality of the Study Based on the NOS Score ^c^	
[35]	Quality of life	Supporting	Randomized against placebo	+++	8	+++
[36]	Depressive symptoms; fatigue	Not supporting	Randomized against placebo	+++	6	++
[37]	Depressive symptoms	Supporting	Prospective with supplementation applied	++	5	++
[38]	Quality of life; fatigue	Supporting	Prospective with supplementation applied	++	6	++
[39]	Depression	Not supporting	Prospective based on self-reporting	+	7	+++
[40]	Quality of life	Supporting	Prospective based on self-reporting	+	6	++

^a^ Conclusions: supporting—major conclusion of the study indicating potential positive influence of vitamin D on mental health of MS patients; not supporting—major conclusion of the study indicating no positive influence of vitamin D on mental health of MS patients; ^b^ Quality of the studies: +++ for studies randomized against placebo; ++ for prospective studies with supplementation applied (intervention); + for prospective studies based on self-reported supplementation; ^c^ Quality of the studies: +++ for low risk of bias (7–9 NOS points); ++ for high risk of bias (4–6 NOS points); + for very high risk of bias (0–3 NOS points).

## Supplementary Materials

The following are available online at https://www.mdpi.com/article/10.3390/nu13124207/s1, Table S1. The applied electronic search strategy for the systematic review of PubMed and Web of Science databases.
