# Supplementary material for: Vitamin D Supplementation and Mental Health in Multiple Sclerosis Patients: A Systematic Review
Source: Nutrients. 2021 Nov 24;13(12):4207. doi: 10.3390/nu13124207 (PMC8705844; doi:10.3390/nu13124207)
Supplement: Supplementary file 1 [file nutrients-13-04207-s001.zip › nutrients-1465028-supplementary.pdf]

nutrients

Systematic Review – supplementary material

The Association Between Vitamin D Supplementation and Mental Health in Multiple Sclerosis Patients: A Systematic Review

Dominika Guzek [1,*], Aleksandra Kołota [2], Katarzyna Lachowicz [2], Dominika Skolmowska [2], Małgorzata Stachoń [2], and Dominika Głąbska [2]

Supplementary Table S1. The applied electronic search strategy for the systematic review of PubMed and Web of Science databases.

Database	Electronic Search Strategy for the Systematic Review
PubMed	(((mental health[Title/Abstract] OR mental disorders[Title/Abstract] OR mental disorder[Title/Abstract] OR psychological distress[Title/Abstract] OR mood disorders[Title/Abstract] OR depression[Title/Abstract] OR suicidal[Title/Abstract] OR suicide[Title/Abstract] OR anxiety[Title/Abstract] OR well-being[Title/Abstract] OR well-being[Title/Abstract] OR quality of life[Title/Abstract] OR self esteem[Title/Abstract] OR self-esteem[Title/Abstract] OR self efficacy[Title/Abstract] OR self-efficacy[Title/Abstract] OR resilience[Title/Abstract] OR empowerment[Title/Abstract] OR social participation[Title/Abstract] OR mental capital[Title/Abstract] OR life skills[Title/Abstract] OR emotional[Title/Abstract] OR psychology[Title/Abstract] OR psychosocial[Title/Abstract] OR psychiatry[Title/Abstract])) AND (vitamin D[Title/Abstract] OR vitamin D2[Title/Abstract] OR vitaminD3[Title/Abstract] OR D2[Title/Abstract] OR D3[Title/Abstract] OR ergocalciferol[Title/Abstract] OR cholecalciferol[Title/Abstract] OR 25-hydroxyvitamin D[Title/Abstract] OR 3-epi-25hydroxyvitaminD[Title/Abstract] OR calcitriol[Title/Abstract] OR dihydroxycholecalciferol[Title/Abstract])) NOT (animal NOT (animal AND human)[MeSH Terms])
Web of Science	(TS=('vitamin D' OR 'vitamin D2' OR 'vitamin D3' OR 'D2' OR 'D3' OR 'ergocalciferol' OR 'cholecalciferol' OR '25-hydroxyvitamin D' OR '3-epi-25 hydroxyvitamin D' OR 'calcitriol' OR 'dihydroxycholecalciferol') AND TS=('mental health' OR 'mental disorders' OR 'mental disorder' OR 'psychological distress' OR 'mood disorder' OR 'depression' OR 'suicidal' OR 'suicide' OR 'anxiety' OR 'well-being' OR 'wellbeing' OR 'quality of life' OR 'self esteem' OR 'self-esteem' OR 'self efficacy' OR 'self-efficacy' OR 'resilience' OR 'empowerment' OR 'social participation' OR 'mental capital' OR 'life skills' OR 'emotional' OR 'psychology' OR 'psychosocial' OR 'psychiatry') NOT TS=('animal' NOT ('animal' AND 'human')))
